# Effect of education on functional network edge efficiency in Alzheimer’s disease

**DOI:** 10.1038/s41598-021-96361-0

**Published:** 2021-08-26

**Authors:** Yeshin Kim, Sung-Woo Kim, Sang Won Seo, Hyemin Jang, Ko Woon Kim, Soo Hyun Cho, Si Eun Kim, Seung Joo Kim, Jin San Lee, Sung Tae Kim, Duk L. Na, Joon-Kyung Seong, Hee Jin Kim

**Affiliations:** 1 Department of Neurology, Kangwon National University Hospital, Kangwon National University College of Medicine, Chuncheon, Republic of Korea; 2 Department of Bio-Convergence Engineering, College of Health Science, Korea University, Seoul, Republic of Korea; 3 Department of Neurology, Samsung Medical Center, Sungkyunkwan University School of Medicine, 50 Ilwon-dong, Gangnam-gu, Seoul, 135-710 Republic of Korea; 4 Department of Health Sciences and Technology, SAIHST, Sungkyunkwan University, Seoul, Republic of Korea; 5 Department of Intelligent Precision Healthcare Convergence, Sungkyunkwan University, Suwon, Republic of Korea; 6 Alzheimer’s Disease Convergence Research Center, Samsung Medical Center, Seoul, Republic of Korea; 7 Department of Neurology, Jeonbuk National University Medical School & Hospital, Jeonju, Korea; 8 Department of Neurology, Chonnam National University Hospital, Gwangju, Korea; 9 Departments of Neurology, Inje University College of Medicine, Haeundae Paik Hospital, Busan, South Korea; 10 Department of Neurology, Gyeongsang National University School of Medicine and Gyeonsang National University Changwon Hospital, Changwon, South Korea; 11 Department of Neurology, Kyung Hee University Hospital, Seoul, South Korea; 12 Department of Radiology, Samsung Medical Center, Sungkyunkwan University School of Medicine, Seoul, Republic of Korea; 13 Stem Cell & Regenerative Medicine Institute, Samsung Medical Center, Seoul, Republic of Korea; 14 Department of Artificial Intelligence, College of Informatics, Korea University, Seoul, Republic of Korea; 15 School of Biomedical Engineering, College of Health Science, Korea University, Seoul, Republic of Korea; 16 Interdisciplinary Program in Precision Public Health, College of Health Science, Korea University, Seoul, Republic of Korea; 17 Department of Digital Health, SAIHST, Sungkyunkwan University, Seoul, Korea

**Keywords:** Cognitive ageing, Cognitive neuroscience

## Abstract

We investigated the effect of education on the edge efficiency in resting state functional networks (RSFNs) in amnestic mild cognitive impairment (aMCI) and Alzheimer’s disease dementia (ADD). We collected the data of 57 early aMCI, 141 late aMCI, 173 mild ADD, and 39 moderate-to-severe ADD patients. We used years of education as a proxy for cognitive reserve. We measured edge efficiency for each edge in RSFNs, and performed simple slope analyses to discover their associations with education level among the four groups. In the late aMCI, a sub-network that had hub nodes in the right middle frontal gyrus and the right posterior cingulate gyrus, showed a positive association between RSFN edge efficiency and education (threshold = 2.5, *p* = 0.0478). There was no negative effect of education on the RSFN edge efficiency. In the early aMCI, mild ADD, and moderate-to-severe ADD, there were no sub-networks showing positive or negative correlation between education and RSFN edge efficiency. There was a positive effect of higher education on RSFN edge efficiency in the late aMCI, but not in the early aMCI or ADD. This indicates that in late aMCI, those who have higher education level have greater ability to resist collapsed functional network.

## Introduction

In the course of Alzheimer’s disease (AD), accumulation of amyloid beta (Aβ) begins 10–15 years before cognitive symptoms appear. Then tau accumulates, followed by synaptic dysfunction and neuronal death. This series of processes lead to cognitive impairment and dementia^1^. However, the degree of cognitive impairment is influenced by individuals’ cognitive reserve^2^. Various pathologic and imaging studies suggested that individuals with higher cognitive reserve (CR) can maintain cognitive function despite having a damaged brain. In AD dementia (ADD), patients with higher CR were reported to be associated with higher Aβ, lower glucose metabolism^3–6^ and more severe cortical atrophy^7–9^. This may imply that patients with higher CR cope better with certain pathologic burdens and maintain their cognition.

Functional magnetic resonance imaging (MRI) has been used in many studies to elucidate the neural mechanism underlying CR. Previous studies reported that RSFN was positively associated with CR in particular areas such as the fronto-parietal control network and default mode network^10–13^.

However, the effect of CR may differ according to disease severity in the course of AD. In preclinical AD, high CR might not be necessary to maintain cognitive function, while in the MCI stage, CR may play an important role in maintaining cognitive function^11,13^. Furthermore, in ADD, the effect of CR might be diminished since the brain is severely damaged.

In this study, we hypothesized that individuals with higher CR cope better with collapsed RSFN edges because they might have reinforced RSFN edge efficiency. In addition, we hypothesized that the effect of CR on reinforced RSFN edge efficiency would differ according to disease severity. In the aMCI stage, when the brain is slightly damaged, individuals with high CR may maximize their ability to reinforce RSFN edge efficiency. However, in ADD, higher CR may not be as useful because reinforcement of RSFN edge efficiency is limited in the moderately damaged brain. Therefore, we investigated the effect of CR on RSFN edge efficiency in each cognitive level: early aMCI, late aMCI, mild ADD, and moderate-to-severe ADD.

## Results

### Demographics

Characteristics of participants are described in Table 1. The mean age of participants did not differ significantly between the four groups. Years of education was higher in the late aMCI group (11.2 ± 4.8) than in the mild ADD group (8.9 ± 5.6). Cognitive function of all domain was worst in patients with moderate-to-severe ADD followed by mild ADD and aMCI. Patients with late aMCI showed worse cognitive function in memory and MMSE score compared to early aMCI patients. The proportion of multiple domain aMCI (50.9% in early aMCI and 61.7% in late aMCI group) was not significantly different between the early and the late aMCI groups.

**Table 1:** Demographic and clinical characteristics of the study population.

	Early aMCI (n = 57)	Late aMCI (n = 141)	Mild ADD (n = 173)	Moderate-to-Severe ADD (n = 39)	*p*-value	Comparison by group^a^
Age, years^b^	69.6 ± 7.9	70.6 ± 8.9	71.2 ± 9.1	72.5 ± 9.6	0.426	
Sex, female^c^	32 (56.1)	82 (58.2)	115 (66.5)	30 (76.9)	0.081	
Education, years^b^	10.4 ± 6.4	11.2 ± 4.8	8.9 ± 5.6	8.6 ± 5.7	0.001	2 > 3
Disease duration, years^b^	2.2 ± 1.9	2.3 ± 1.9	3.6 ± 2.1	6.2 ± 3.2	< 0.001	1, 2 < 3 < 4
**Neuropsychological test**						
Language function^b^	41.4 ± 1.1	40.6 ± 0.8	31.2 ± 11.5	23.5 ± 12.7	< 0.001	1, 2 > 3 > 4
Visuospatial function^b^	29.3 ± 0.9	29.3 ± 0.6	22.5 ± 10.1	13.5 ± 12.5	< 0.001	1, 2 > 3 > 4
Memory function^b^	41.7 ± 1.1	35.5 ± 0.7	27.7 ± 6.4	22.2 ± 5.2	< 0.001	1 > 2 > 3 > 4
Frontal function^b^	42.3 ± 2.7	38.0 ± 1.4	28.5 ± 18.6	22.1 ± 26.5	< 0.001	1, 2 > 3, 4
MMSE^b^	26.9 ± 2.7	25.7 ± 2.8	20.4 ± 4.2	13.7 ± 5.2	< 0.001	1 > 2 > 3 > 4

*aMCI* amnestic mild cognitive impairment, *ADD* Alzheimer’s disease dementia, *MMSE* Mini-Mental State Examination.

^a^1, Early aMCI; 2, Late aMCI; 3, Mild ADD; 4, Moderate-to-severe ADD.

^b^Continuous variables are expressed as mean ± standard deviation.

^c^Dichotomous variables are expressed as number (relative frequency).

### Differences of gray matter volume in Alzheimer’s disease spectrum

We analyzed gray matter volume of late aMCI, mild AD, and moderate to severe AD in comparison to early aMCI group (Supplementary Table 1). There was no difference of gray matter volume between early and late aMCI group. However, in mild and moderate-to-severe AD groups, most of the regions showed decreased volume in comparison to early aMCI group.

### Effect of education on RSFN edge efficiency in Alzheimer’s disease spectrum

**Figure 1:**
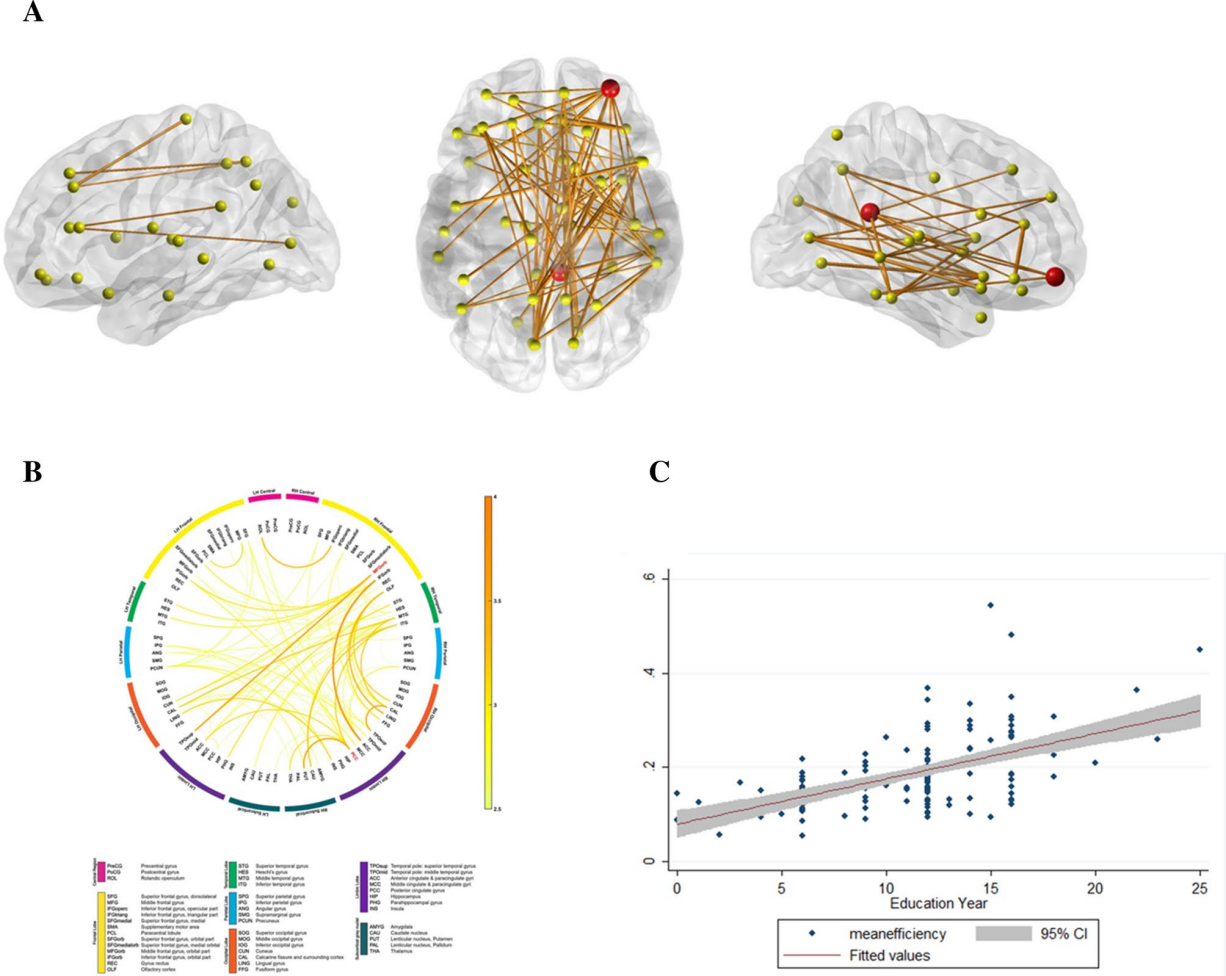
(**A**) In the late aMCI group, a sub-network showed a positive correlation between years of education and RSFN edge efficiency. The red circles represent the hub regions, which are located in the orbital part of the right middle frontal gyrus (MFGorb), and the right posterior cingulate gyrus (PCC). (**B**) Connectogram showing the sub-network. The thickness and colors represents the t-statistics computed from simple slope analyses. Hub regions are presented in red color. (**C**) Correlation between education and mean RSFN edge efficiency of the sub-network in the late aMCI group. *aMCI* amnestic mild cognitive impairment.

Figure 1 shows the identified sub-networks whose edges had positive correlations between years of education and RSFN edge efficiency in late aMCI (threshold = 2.5, *p* = 0.0478). The hub nodes that were positively correlated with education were the orbital part of the right middle frontal gyrus and the right posterior cingulate gyrus (Fig. 1A, denoted by red circles). Sub-networks were distributed across the frontal, parietal, and temporal lobes. Detailed information regarding these sub-networks is described in Fig. 1B. There were no sub-networks that showed a negative correlation between education and RSFN edge efficiency. Further analysis showed that education had positive correlation with mean RSFN edge efficiency of the sub-network in the late aMCI group (β = 33.9, p < 0.001) (Fig. 1C).

In the early aMCI, mild ADD, and moderate-to-severe ADD groups, there were no sub-networks that showed positive or negative correlations between education and RSFN edge efficiency.

## Discussion

In this study, we investigated the effect of education on the RSFN edge efficiency in patients with aMCI and ADD. Higher educational level was positively associated with the RSFN edge efficiency in the late aMCI stage. We did not observe negative effects of education on the RSFN edge efficiency. In early aMCI, mild ADD, and moderate-to-severe ADD, however, there were no significant sub-networks showing positive or negative correlations with education. The hubs of sub-networks that were positively correlated with education in the late aMCI group were the right middle frontal gyrus and the right posterior cingulate gyrus. Thus, we suggest that in late aMCI stage, those who have higher education level could have greater ability to resist collapsed functional network.

First, we found that the effect of education on the RSFN edge efficiency differed based on cognitive stage. The effect of education on the RSFN edge efficiency was significant only in the late aMCI group, but not in the early aMCI, mild ADD, or moderate-to-severe ADD group. This finding is consistent with previous studies showing that the difference in local topological properties of functional connectivity according to CR was significant only in MCI, but not in ADD^13^. According to previous studies, patients in the early and late stages of MCI showed different clinical characteristics, including dementia conversion rate and brain networks^14–16^. Furthermore, the effects of education on the progression of aMCI in the early and late stages were different^17^. However, there is a lack of previous studies on whether the relationship between CR and topological properties of functional connectivity differ in early and late MCI stage. Looking at our results for the early and late aMCI stages, it can be suggested that the effect of CR on the RSFN edge efficiency appears when the pathologic burden reaches a certain level, not in the very early stage, but in the late aMCI stage. Having higher RSFN edge efficiency in patients with higher CR means they could resist collapsed edges better than those patients with lower CR. Even though there will be network disruption as AD progresses, the ability to resist a collapsed network via increased RSFN edge efficiency is assumed to be beneficial with respect to maintaining cognitive function. However, after the late aMCI stage, when network disruption is more severe, it overwhelms the effect of CR to resist collapsed networks.

Second, we found that high CR was associated with the RSFN edge efficiency in specific regions: the right middle frontal gyrus and right posterior cingulate gyrus. The functional network has been investigated as neural substrate of CR in many studies. Although the laterality differed among the studies, the fronto-parietal network was reported to be related to high CR, which was also confirmed in our study^13,18–20^. One study reported that bilateral fronto-parietal networks were associated with CR^13^ while Franzmeier et al. reported that the left frontal network could be the neural substrate of CR^18–20^. The fronto-parietal network is known to be involved in various tasks through its regulation of cognitive control abilities^21,22^. It is described as a flexible hub for cognitive control which can change connectivity associated with other networks and can adapt task demands^22,23^. A previous study reported that the fronto-parietal network has inter-individual variability^24^. This supports our hypothesis that high CR could affect the edge efficiency of specific hubs, especially flexible hubs in the fronto-parietal network. This may allow patients to resist the collapse of the RSFN edges in the early stage of the AD process (aMCI stages).

Our study has some limitations. First, patients were diagnosed based on clinical criteria and we did not confirm amyloid or tau biomarkers. Second, we used years of education as a proxy for CR, but this cannot capture occupational, social, or leisure experiences. However, even though there is ongoing controversy about the appropriate proxy for CR, years of education is a widely-used proxy^25,26^. Third, there were fewer participants in the early aMCI and moderate-to-severe ADD groups which might have led to non-significant findings in these groups. Fourth, we did not analyze the effect of education on RSFN edge efficiency in subjects with normal cognition, which needs to be explored in further studies. Nevertheless, our study provided additional evidence that beneficial effects of CR are observed in late aMCI stage patients.

In conclusion, we showed that a higher education level was associated with a stronger impact on the RSFN edge efficiency in the fronto-parietal network. The fronto-parietal network might be an important network allowing patients to cope with cognitive decline in the late aMCI stage.

## Methods

### Subjects

We collected data from 245 aMCI and 301 AD patients who underwent rsfMRI from January 2008 to December 2010 at Samsung Medical Center.

Patients with aMCI met Petersen’s criteria^27^ with the following modifications: (1) subjective memory complaint by the patient or his/her caregiver, (2) normal activities of daily living (ADL) as judged by both an interview with a clinician and the standardized ADL scale as previously described^28^, and (3) objective verbal or visual memory impairment below − 1.0 standard deviations (SD) of age- and education matched norms on neuropsychological tests^29^. Among them, patients were classified into early aMCI when their delayed recall scores for either visual or verbal memory tests were between − 1.5 to − 1.0 SD and none were below − 1.5 SD of the norms. Patients were classified into late aMCI when their delayed recall scores for either visual or verbal memory tests were below − 1.5 SD of the norms^17^. ADD was diagnosed when patients met diagnostic criteria for probable ADD according to the National Institute of Neurological and Communicative Disorders and Stroke and the AD and Related Disorders Association^30^. Mild ADD was defined as Clinical Dementia Rating (CDR) scale of 0.5 or 1, and moderate-to-severe ADD was defined as CDR ≥ 2. We excluded 30 patients with severe white matter hyperintensities (WMH), defined as deep WMH ≥ 25 mm and periventricular WMH ≥ 10 mm. We also excluded 23 patients with history of traumatic brain injury, cortical stroke, seizure, brain surgery, and current systemic medical disease that could affect cognition and 83 patients whose RSFN analysis was unavailable due to head motion.

We finally analyzed 57 early aMCI, 141 late aMCI, 173 mild ADD, and 39 moderate-to-severe ADD patients.

This study was approved by the Institutional Review Board of Samsung Medical Center and the methods were carried out in accordance with the relevant guidelines and regulations. This manuscript does not contain information or image that can lead to identification of a study participant. The requirement for participant’s informed consent was waived by the Institutional Review Board of Samsung Medical Center since we used retrospective de-identified data.

### Neuropsychological tests

All subjects underwent detailed clinical interviews, neurologic examinations, and detailed neuropsychological tests. We determined patients’ cognitive status according to the results of a standardized neuropsychological battery called the Seoul Neuropsychological Screening Battery^29,31,32^. We used composite scores as followings^29,33^. The language function was derived from the Boston naming test (range 0–60). The visuospatial function was derived from the Rey Complex Figure Test (0–36). The memory score was calculated by summing the following memory scores: verbal memory tests (Seoul Verbal Learning Test immediate recall, delayed recall, and recognition score) and visual memory tests (Rey Complex Figure Test immediate recall, delayed recall, and recognition score; 0–144). The frontal-executive score was calculated by summing scores of the category word generation test, phonemic word generation test, and Stroop color reading test (0–55). Global cognitive function was assessed using the MMSE.

### Image acquisition

Both structural and functional images were acquired at Samsung Medical Center using a Philips 3.0-T Intera Achieva MRI scanner (Philips Medical Systems, Best, the Netherlands) as previously described^34^.

### Image preprocessing

The rsfMRI data were preprocessed using FEAT (FMRI Expert Analysis Tool) in FSL 5.0 (FMIRB’s Software Library)^35^. The first three volumes were discarded to allow for stabilization of MR signals. We then conducted the following preprocessing procedures: motion correction using MCFLIRT, slice-timing correction using sinc interpolation with a Hanning windowing kernel, removal of non-brain regions using BET and grand-mean scaling for removing intersession variance in the global signal. For the motion correction process, gross head motion was defined as relative mean displacement > 0.55 mm^36,37^, and 83 subjects (22 aMCI and 61 AD) with gross head motion were initially excluded from this experiment. In addition, the non-neural fluctuations including white matter (WM) and cerebrospinal fluid (CSF) signals^38,39^, and the 24 motion parameters derived by head motion correction^40^ were regressed out. The WM and CSF signals were extracted from the partial volume map by thresholding 0.9 using FSL-FAST.

The volumetric regions of interest (ROIs) were defined according to the Automated Anatomical Labeling (AAL) brain atlas^41^, which include 40 cerebral cortical regions and five subcortical regions for each hemisphere. For transformations between the atlas image and structural images, and between structural and functional images, non-linear registration and boundary-based registration were performed, respectively. For every subject, blood-oxygenation-level-dependent (BOLD) signals over all voxels in each region were averaged, resulting in 90 regional mean BOLD signals.

T1-weighted MR images were preprocessed using FreeSurfer v5.1.0^42^ to obtain intracranial volume (ICV), and gray matter volumes for the 90 regions.

### Resting-state functional network construction

RSFNs were constructed by measuring pair-wise similarity between two regional BOLD signals using wavelet correlation^43–48^. The wavelet analysis is more advantageous for dealing with rsfMRI which has a long memory in time or a $1/f$ power spectrum in the frequency domain rather than with correlation or coherence analysis^48–50^. Specifically, we first performed wavelet transformation on 90 time-series using maximum overlap discrete wavelet transform (MODWT) with a fourth-order Daubechiese wavelet filter (db8) and a reflection boundary condition. We chose this wavelet filter because it was found to provide good decorrelation of wavelet detail coefficients^45,51^, although the type of wavelet filter has been found to have little influence on fMRI data analysis^44^. In addition, we chose the reflection boundary condition because it is appropriate to time-series that have a small number of time points (in our case, 97 time points)^45,46^. According to previous studies that showed low frequency signals were more associated with the AD continuum^52,53^, we extracted scale 4 wavelet coefficients (approximately 0.01–0.02 Hz) out of the first four wavelet scales using the Wavelet Toolbox in Matlab R2016a^54,55^. We then calculated wavelet correlation coefficients on all possible $$\left(\begin{array}{c}90\\ 2\end{array}\right)=4005$$ pairs of 90 regional mean BOLD signals^43,56^ using the WMTSA Wavelet Toolkit in Matlab^55^.

We constructed RSFNs with only positive coefficients since anti-correlations are hard to quantify and interpret in terms of network topological measures^57^.

### Edge efficiency

**Figure 2:**
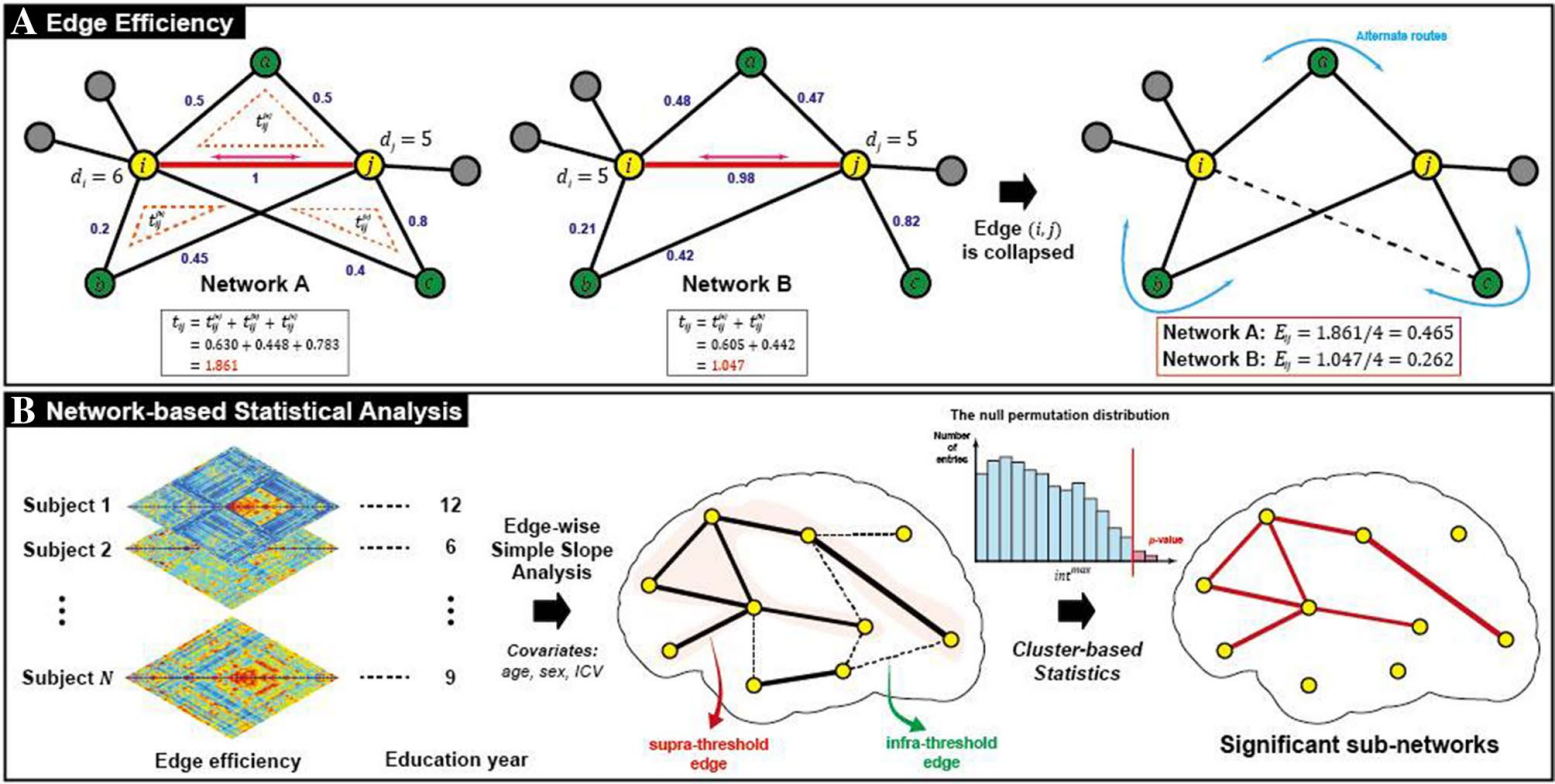
Schematic overview of the proposed method. (**A**) The concept of edge efficiency is depicted with an example. In this example, edges in network A and network B are same, except that the edge (i, c) does not exist in the network B. Although the exchanges of information through the edge (i, j) (red line and magenta arrow) are similar in both networks, different network topology results in different edge efficiency values for the edge (i, j) in the two networks. When the edge (i, j) is removed, information communicating through other paths alternative to the edge (i, j) is different in network A and B. (**B**) Network-based statistical analysis to identify sub-networks whose edge efficiency values are significantly associated with education years. The analysis was performed using cluster-based statistics. Note that it detects significant sub-networks, and it cannot extract any single significant edge such as the edge (i, c) in network A.

Efficiency in brain network science is a measure of how efficiently information is exchanged^58^, and efficiency can be defined either globally or locally. Local efficiency quantifies the transformation of information in a part of the network. The local efficiency at the node-level, for example, indicates the efficiency between two nodes^57,58^. Similarly, we can define the local efficiency at the edge-level that represents the efficiency in exchange of information through a network edge, which can be quantified to the extent that information flows through a neighborhood system when an edge is removed. Specifically, we computed the local efficiency at the edge $\left(i,j\right)$ of RSFNs, or an edge efficiency (${E}_{\mathrm{loc},ij})$, as follows:

$${E}_{\mathrm{loc},ij}=\frac{{t}_{ij}}{\mathrm{min}\left({d}_{i}-1,{d}_{j}-1\right)},$$

where ${t}_{ij}$ denotes the sum of geometric means of three sides of triangles that included an edge $\left(i,j\right)$, ${d}_{i}$ and ${d}_{j}$ are degrees of nodes incident to the edge (Fig. 2A)^59–61^. The triangles in the nominator quantify other paths that replace the removed edge. Thus, the edge efficiency can be interpreted as an edge’s characteristic ability to resist collapse and make the entire network function as before.

### Statistical analysis

Demographic and clinical characteristics of the study population are presented as continuous or categorical variables. To test difference between groups, we used one-way analysis of variance (ANOVA) for continuous variables or the Chi-square test followed by the Bonferroni method. To test differences of cognitive function between the groups, analysis of covariance (ANCOVA) was used, controlling for age and education followed by the Bonferroni method. Statistical analyses were performed with STATA version 14 (StataCorp LP, College Station, TX, USA). Two-tailed *p*-values less than 0.05 were considered statistically significant.

To test difference in gray matter volume of each group, we used two-sample (Student’s or Welch’s) *t*-test between early aMCI and late aMCI, between early aMCI and mild AD, and between early aMCI and moderate-to-severe AD, respectively. For multiple comparison correction, we performed false discovery rate (FDR) procedure over 90 regions^62^.

To discover whether associations exist between CR and ${E}_{\mathrm{loc},ij}$ in the four groups for each edge $\left(i,j\right)$, we performed simple slope analyses with a multiple linear regression equation^63^ We used the years of education as a proxy for CR. For each edge, we formulated a linear regression equation with ${E}_{\mathrm{loc},ij}$ as a dependent variable, the year of education and group as independent variables. One-way interactions between the groups and the years of education were added to the equation. Age, sex, and ICV were also added to the equation as covariates. Simple slope coefficients were transformed to *t*-statistics.

For statistical significance tests and multiple comparison correction, we performed cluster-based statistics (CBS) analysis with simple slope coefficients (Fig. 2B). The CBS analysis tests statistical hypotheses at the level of sub-networks, not of edges, and identifies significant sub-networks as connected components^47,64^. For each group, the *p* values of sub-networks with supra-threshold edges were estimated from the empirical null distribution for *N* statistics of sub-networks generated based on a permutation procedure^65,66^. For the statistics, we used the maximum values of the sum of *t*-statistics among all connected components for each permutation, denoted as ${int}^{max}$. In this study, we set *N* and the significance level to 10,000, and 0.05, respectively. We repeated these procedures with different thresholds that ranged from − 3.5 to − 1.5 and 1.5 to 3.5 in units of 0.1, and chose one with the highest absolute value among those that produced significant results.

Finally, we identified hub regions from the sub-networks with the nodes whose modified z scores of nodal degrees were larger than two standard deviations, so that they signified the regions of most influence in the sub-networks.

## Supplementary Information

Supplementary Table S1.

## References

[1] Sperling RA, Aisen PS, Beckett LA (2011). Toward defining the preclinical stages of Alzheimer's disease: Recommendations from the National Institute on Aging-Alzheimer's Association workgroups on diagnostic guidelines for Alzheimer's disease. Alzheimer's Dement..

[2] Stern Y (2012). Cognitive reserve in ageing and Alzheimer's disease. Lancet Neurol..

[3] Alexander GE, Furey ML, Grady CL (1997). Association of premorbid intellectual function with cerebral metabolism in Alzheimer's disease: Implications for the cognitive reserve hypothesis. Am. J. Psychiatry.

[4] Kemppainen NM, Aalto S, Karrasch M (2008). Cognitive reserve hypothesis: Pittsburgh Compound B and fluorodeoxyglucose positron emission tomography in relation to education in mild Alzheimer's disease. Ann. Neurol..

[5] Ewers M, Insel PS, Stern Y, Weiner MW (2013). Cognitive reserve associated with FDG-PET in preclinical Alzheimer disease. Neurology.

[6] Morbelli S, Perneczky R, Drzezga A (2013). Metabolic networks underlying cognitive reserve in prodromal Alzheimer disease: A European Alzheimer disease consortium project. J. Nucl. Med..

[7] Cho H, Jeon S, Kim C (2015). Higher education affects accelerated cortical thinning in Alzheimer's disease: A 5-year preliminary longitudinal study. Int. Psychogeriatr..

[8] Seo SW, Im K, Lee JM (2011). Effects of demographic factors on cortical thickness in Alzheimer's disease. Neurobiol. Aging.

[9] Liu Y, Julkunen V, Paajanen T (2012). Education increases reserve against Alzheimer's disease—Evidence from structural MRI analysis. Neuroradiology.

[10] Franzmeier N, Buerger K, Teipel S, Stern Y, Dichgans M, Ewers M (2017). Cognitive reserve moderates the association between functional network anti-correlations and memory in MCI. Neurobiol. Aging.

[11] Franzmeier N, Caballero MA, Taylor A (2016). Resting-state global functional connectivity as a biomarker of cognitive reserve in mild cognitive impairment. Brain Imaging Behav..

[12] Bozzali M, Dowling C, Serra L (2015). The impact of cognitive reserve on brain functional connectivity in Alzheimer's disease. J. Alzheimer's Dis. JAD.

[13] Serra L, Mancini M, Cercignani M (2017). Network-based substrate of cognitive reserve in Alzheimer's disease. J. Alzheimer's Dis. JAD.

[14] Edmonds EC, McDonald CR, Marshall A (2019). Early versus late MCI: Improved MCI staging using a neuropsychological approach. Alzheimer's Dement..

[15] Jessen F, Wolfsgruber S, Wiese B (2014). AD dementia risk in late MCI, in early MCI, and in subjective memory impairment. Alzheimer's Dement..

[16] Aisen PS, Petersen RC, Donohue MC (2010). Clinical core of the Alzheimer's disease neuroimaging initiative: Progress and plans. Alzheimer's Dement..

[17] Ye BS, Seo SW, Cho H (2013). Effects of education on the progression of early-versus late-stage mild cognitive impairment. Int. Psychogeriatr..

[18] Franzmeier N, Düzel E, Jessen F (2018). Left frontal hub connectivity delays cognitive impairment in autosomal-dominant and sporadic Alzheimer's disease. Brain J. Neurol..

[19] Franzmeier N, Hartmann J, Taylor ANW (2018). The left frontal cortex supports reserve in aging by enhancing functional network efficiency. Alzheimer's Res. Ther..

[20] Franzmeier N, Hartmann JC, Taylor ANW (2017). Left frontal hub connectivity during memory performance supports reserve in aging and mild cognitive impairment. J. Alzheimer's Dis. JAD.

[21] Dosenbach NU, Fair DA, Cohen AL, Schlaggar BL, Petersen SE (2008). A dual-networks architecture of top-down control. Trends Cogn. Sci..

[22] Zanto TP, Gazzaley A (2013). Fronto-parietal network: Flexible hub of cognitive control. Trends Cogn. Sci..

[23] Cole MW, Reynolds JR, Power JD, Repovs G, Anticevic A, Braver TS (2013). Multi-task connectivity reveals flexible hubs for adaptive task control. Nat. Neurosci..

[24] Marek S, Dosenbach NUF (2018). The frontoparietal network: Function, electrophysiology, and importance of individual precision mapping. Dialogues Clin. Neurosci..

[25] Serra L, Cercignani M, Petrosini L (2011). Neuroanatomical correlates of cognitive reserve in Alzheimer disease. Rejuvenation Res..

[26] Staekenborg SS, Kelly N, Schuur J (2020). Education as proxy for cognitive reserve in a large elderly memory clinic: 'Window of Benefit'. J. Alzheimer's Dis. JAD.

[27] Petersen RC, Smith GE, Waring SC, Ivnik RJ, Tangalos EG, Kokmen E (1999). Mild cognitive impairment: Clinical characterization and outcome. Arch. Neurol..

[28] Ku HM, Kim JH, Kwon EJ (2004). A study on the reliability and validity of Seoul-Instrumental Activities of Daily Living (S-IADL). J. Korean Neuropsychiatr. Assoc..

[29] Ahn H-J, Chin J, Park A (2010). Seoul Neuropsychological Screening Battery-dementia version (SNSB-D): A useful tool for assessing and monitoring cognitive impairments in dementia patients. J. Korean Med. Sci..

[30] McKhann GM, Knopman DS, Chertkow H (2011). The diagnosis of dementia due to Alzheimer's disease: Recommendations from the National Institute on Aging-Alzheimer's Association workgroups on diagnostic guidelines for Alzheimer's disease. Alzheimer's Dement..

[31] Kang Y, Na D, Hahn S (2003). Seoul Neuropsychological Screening Battery.

[32] Kang SH, Park YH, Lee D (2019). The cortical neuroanatomy related to specific neuropsychological deficits in Alzheimer's continuum. Dement. Neurocogn. Disord..

[33] Kim HJ, Park S, Cho H (2018). Assessment of extent and role of tau in subcortical vascular cognitive impairment using 18F-AV1451 positron emission tomography imaging. JAMA Neurol..

[34] Kim HJ, Cha J, Lee J-M (2016). Distinctive resting state network disruptions among Alzheimer’s disease, subcortical vascular dementia, and mixed dementia patients. J. Alzheimers Dis..

[35] Jenkinson M, Beckmann CF, Behrens TE, Woolrich MW, Smith SM (2012). Fsl. Neuroimage.

[36] Satterthwaite TD, Wolf DH, Loughead J (2012). Impact of in-scanner head motion on multiple measures of functional connectivity: Relevance for studies of neurodevelopment in youth. Neuroimage.

[37] Satterthwaite TD, Elliott MA, Gerraty RT (2013). An improved framework for confound regression and filtering for control of motion artifact in the preprocessing of resting-state functional connectivity data. Neuroimage.

[38] Dagli MS, Ingeholm JE, Haxby JV (1999). Localization of cardiac-induced signal change in fMRI. Neuroimage.

[39] Weissenbacher A, Kasess C, Gerstl F, Lanzenberger R, Moser E, Windischberger C (2009). Correlations and anticorrelations in resting-state functional connectivity MRI: A quantitative comparison of preprocessing strategies. Neuroimage.

[40] Friston KJ, Williams S, Howard R, Frackowiak RS, Turner R (1996). Movement-related effects in fMRI time-series. Magn. Reson. Med..

[41] Tzourio-Mazoyer N, Landeau B, Papathanassiou D (2002). Automated anatomical labeling of activations in SPM using a macroscopic anatomical parcellation of the MNI MRI single-subject brain. Neuroimage.

[42] Dale AM, Fischl B, Sereno MI (1999). Cortical surface-based analysis: I. Segmentation and surface reconstruction. Neuroimage.

[43] Achard S, Salvador R, Whitcher B, Suckling J, Bullmore E (2006). A resilient, low-frequency, small-world human brain functional network with highly connected association cortical hubs. J. Neurosci..

[44] Zhang Z, Telesford QK, Giusti C, Lim KO, Bassett DS (2016). Choosing wavelet methods, filters, and lengths for functional brain network construction. PLoS One.

[45] Patel AX, Bullmore ET (2016). A wavelet-based estimator of the degrees of freedom in denoised fMRI time series for probabilistic testing of functional connectivity and brain graphs. Neuroimage.

[46] Supekar K, Menon V, Rubin D, Musen M, Greicius MD (2008). Network analysis of intrinsic functional brain connectivity in Alzheimer's disease. PLoS Comput. Biol..

[47] Zalesky A, Fornito A, Bullmore ET (2010). Network-based statistic: Identifying differences in brain networks. Neuroimage.

[48] Achard S, Bullmore E (2007). Efficiency and cost of economical brain functional networks. PLoS Comput. Biol..

[49] Maxim V, Şendur L, Fadili J (2005). Fractional Gaussian noise, functional MRI and Alzheimer's disease. Neuroimage.

[50] Wink AM, Bernard F, Salvador R, Bullmore E, Suckling J (2006). Age and cholinergic effects on hemodynamics and functional coherence of human hippocampus. Neurobiol. Aging.

[51] Bullmore E, Long C, Suckling J (2001). Colored noise and computational inference in neurophysiological (fMRI) time series analysis: Resampling methods in time and wavelet domains. Hum. Brain Mapp..

[52] Yang L, Yan Y, Li Y (2020). Frequency-dependent changes in fractional amplitude of low-frequency oscillations in Alzheimer’s disease: a resting-state fMRI study. Brain Imaging Behav..

[53] Li Y, Yao H, Lin P (2017). Frequency-dependent altered functional connections of default mode network in Alzheimer’s disease. Front. Aging Neurosci..

[54] Percival DB, Walden AT (2006). Wavelet Methods for Time Series Analysis.

[55] Percival DB, Mofjeld HO (1997). Analysis of subtidal coastal sea level fluctuations using wavelets. J. Am. Stat. Assoc..

[56] Whitcher B, Guttorp P, Percival DB (2000). Wavelet analysis of covariance with application to atmospheric time series. J. Geophys. Res. Atmos..

[57] Rubinov M, Sporns O (2010). Complex network measures of brain connectivity: Uses and interpretations. Neuroimage.

[58] Latora V, Marchiori M (2001). Efficient behavior of small-world networks. Phys. Rev. Lett..

[59] Radicchi F, Castellano C, Cecconi F, Loreto V, Parisi D (2004). Defining and identifying communities in networks. Proc. Natl. Acad. Sci..

[60] Wang J, Li M, Wang H, Pan Y (2011). Identification of essential proteins based on edge clustering coefficient. IEEE ACM Trans. Comput. Biol. Bioinf..

[61] Saramäki J, Kivelä M, Onnela J-P, Kaski K, Kertesz J (2007). Generalizations of the clustering coefficient to weighted complex networks. Phys. Rev. E.

[62] Benjamini Y, Hochberg Y (1995). Controlling the false discovery rate: A practical and powerful approach to multiple testing. J. R. Stat. Soc. Ser. B (Methodol.).

[63] Cohen J, Cohen P, West SG, Aiken LS (2013). Applied Multiple Regression/Correlation Analysis for the Behavioral Sciences.

[64] Han CE, Yoo SW, Seo SW, Na DL, Seong J-K (2013). Cluster-based statistics for brain connectivity in correlation with behavioral measures. PLoS One.

[65] Freedman D, Lane D (1983). A nonstochastic interpretation of reported significance levels. J. Bus. Econ. Stat..

[66] Winkler AM, Ridgway GR, Webster MA, Smith SM, Nichols TE (2014). Permutation inference for the general linear model. Neuroimage.

