# Supplementary material for: Effect of education on functional network edge efficiency in Alzheimer’s disease
Source: Sci Rep. 2021 Aug 26;11:17255. doi: 10.1038/s41598-021-96361-0 (PMC8390462; doi:10.1038/s41598-021-96361-0)
Supplement: Supplementary file 1 — Supplementary Table S1. [file 41598_2021_96361_MOESM1_ESM.docx]

**sTable 1. Comparison of volume of ROIs of early aMCI group and other subgroups**

|  | **Late aMCI vs Early aMCI** | |  | **Mild AD vs Early aMCI** | |  | **Moderate-to-severe AD vs Early aMCI** | |
| --- | --- | --- | --- | --- | --- | --- | --- | --- |
| **Regions** | ***t* statistics** | **FDR-corrected *p*** |  | ***t* statistics** | **FDR-corrected *p*** |  | ***t* statistics** | **FDR-corrected *p*** |
| Left Precentral gyrus | -2.67552 | 0.107382 |  | **-4.04887** | **0.000181** |  | **-4.80647** | **1.38E-05** |
| Right Precentral gyrus | -0.57704 | 0.683902 |  | **-2.58103** | **0.012743** |  | **-3.43896** | **0.001075** |
| Left Superior frontal gyrus, dorsolateral | -2.67472 | 0.107382 |  | **-5.22436** | **6.79E-06** |  | **-5.73972** | **5.47E-07** |
| Right Superior frontal gyrus, dorsolateral | -1.98778 | 0.173623 |  | **-4.57735** | **3.17E-05** |  | **-6.48692** | **3.37E-08** |
| Left Superior frontal gyrus, orbital part | -1.5114 | 0.269077 |  | **-3.12908** | **0.002832** |  | **-3.55372** | **0.000771** |
| Right Superior frontal gyrus, orbital part | -1.53906 | 0.269077 |  | **-3.26516** | **0.002029** |  | **-3.25081** | **0.001892** |
| Left Middle frontal gyrus | -1.77156 | 0.219437 |  | **-5.56567** | **5.48E-07** |  | **-5.65471** | **7.16E-07** |
| Right Middle frontal gyrus | -1.72521 | 0.227821 |  | **-5.03586** | **5.44E-06** |  | **-4.61687** | **2.85E-05** |
| Left Middle frontal gyrus, orbital part | -0.95081 | 0.514304 |  | **-3.05821** | **0.003452** |  | **-4.19776** | **0.000108** |
| Right Middle frontal gyrus, orbital part | -0.5766 | 0.683902 |  | **-2.31133** | **0.024119** |  | **-3.06978** | **0.003111** |
| Left Inferior frontal gyrus, opercular part | -1.24565 | 0.364039 |  | **-3.06969** | **0.003379** |  | **-4.04522** | **0.000172** |
| Right Inferior frontal gyrus, opercular part | -1.22121 | 0.375645 |  | **-4.00089** | **0.0003** |  | **-3.94244** | **0.000229** |
| Left Inferior frontal gyrus, triangular part | 0.651727 | 0.653249 |  | -1.95648 | 0.055317 |  | **-3.48889** | **0.000925** |
| Right Inferior frontal gyrus, triangular part | -0.2008 | 0.880179 |  | **-2.23387** | **0.031042** |  | **-3.11972** | **0.002774** |
| Left Inferior frontal gyrus, orbital part | -1.40892 | 0.300831 |  | **-3.73247** | **0.000469** |  | **-4.96162** | **8.17E-06** |
| Right Inferior frontal gyrus, orbital part | -0.07254 | 0.95283 |  | **-2.35569** | **0.021754** |  | **-4.02196** | **0.000181** |
| Left Rolandic operculum | -0.12467 | 0.921384 |  | **-2.46694** | **0.016574** |  | **-2.94741** | **0.004436** |
| Right Rolandic operculum | -0.93789 | 0.515588 |  | **-3.29257** | **0.001883** |  | **-3.7746** | **0.00039** |
| Left Supplementary motor area | -2.54815 | 0.116832 |  | **-4.33168** | **0.000115** |  | **-5.19038** | **3.88E-06** |
| Right Supplementary motor area | -1.81956 | 0.218334 |  | **-4.27847** | **8.60E-05** |  | **-5.46079** | **1.39E-06** |
| Left Olfactory cortex | -1.55488 | 0.269077 |  | **-3.0179** | **0.004571** |  | **-3.37922** | **0.001289** |
| Right Olfactory cortex | -0.57045 | 0.683902 |  | -1.05353 | 0.305601 |  | -1.63795 | 0.10596 |
| Left Superior frontal gyrus, medial | -1.04332 | 0.454708 |  | **-4.16542** | **0.000195** |  | **-5.60229** | **8.21E-07** |
| Right Superior frontal gyrus, medial | -0.42722 | 0.770161 |  | **-3.48372** | **0.001046** |  | **-4.46825** | **4.40E-05** |
| Left Superior frontal gyrus, medial orbital part | -1.79718 | 0.219437 |  | **-2.89517** | **0.005346** |  | **-4.06172** | **0.000168** |
| Right Superior frontal gyrus, medial orbital part | -1.83231 | 0.218334 |  | **-3.32224** | **0.001733** |  | **-4.42849** | **5.01E-05** |
| Left Gyrus rectus | -1.7778 | 0.219437 |  | **-4.47914** | **4.44E-05** |  | **-4.9558** | **8.17E-06** |
| Right Gyrus rectus | -1.18243 | 0.390222 |  | **-2.63825** | **0.010982** |  | **-3.10951** | **0.002826** |
| Left Insula | -2.5072 | 0.116832 |  | **-5.95179** | **8.93E-08** |  | **-7.35784** | **1.27E-09** |
| Right Insula | -1.94484 | 0.184241 |  | **-6.64404** | **6.65E-09** |  | **-7.8273** | **2.20E-10** |
| Left Anterior cingulate & paracingulate gyri | -2.72376 | 0.107382 |  | **-4.53146** | **3.70E-05** |  | **-4.48498** | **4.30E-05** |
| Right Anterior cingulate & paracingulate gyri | -1.52326 | 0.269077 |  | -1.72173 | 0.094149 |  | -1.80178 | 0.077364 |
| Left Middle cingulate & paracingulate gyri | -1.82325 | 0.218334 |  | **-5.29529** | **1.68E-06** |  | **-6.02402** | **1.97E-07** |
| Right Middle cingulate & paracingulate gyri | -1.74527 | 0.225014 |  | **-4.18383** | **0.000112** |  | **-4.47992** | **4.30E-05** |
| Left Posterior cingulate gyrus | -2.70243 | 0.107382 |  | **-3.62791** | **0.000905** |  | **-3.52157** | **0.000842** |
| Right Posterior cingulate gyrus | -2.21628 | 0.150275 |  | **-2.54634** | **0.014973** |  | **-2.32436** | **0.024137** |
| Left Hippocampus | -0.74834 | 0.611396 |  | **-2.09571** | **0.042915** |  | **-4.04184** | **0.000172** |
| Right Hippocampus | -1.50265 | 0.269077 |  | **-3.22577** | **0.002699** |  | **-4.55419** | **3.46E-05** |
| Left Parahippocampal gyrus | -3.08202 | 0.090638 |  | **-6.31934** | **2.05E-08** |  | **-8.6581** | **1.18E-11** |
| Right Parahippocampal gyrus | -1.52448 | 0.269077 |  | **-5.8481** | **1.40E-07** |  | **-7.07258** | **3.00E-09** |
| Left Amygdala | -0.70711 | 0.631591 |  | -0.78785 | 0.442901 |  | -1.64704 | 0.105232 |
| Right Amygdala | 0.05093 | 0.959434 |  | 1.549684 | 0.128307 |  | 0.513398 | 0.608877 |
| Left Calcarine fissure and surrounding cortex | -1.36217 | 0.320891 |  | **-3.88712** | **0.000292** |  | **-3.98809** | **0.000201** |
| Right Calcarine fissure and surrounding cortex | -1.51803 | 0.269077 |  | **-3.16122** | **0.002676** |  | **-3.12029** | **0.002774** |
| Left Cuneus | -0.58335 | 0.683902 |  | **-3.77528** | **0.000417** |  | **-3.89487** | **0.000267** |
| Right Cuneus | -0.7532 | 0.611396 |  | **-3.91913** | **0.000264** |  | **-3.07045** | **0.003111** |
| Left Lingual gyrus | -1.33602 | 0.329566 |  | **-3.42469** | **0.001239** |  | **-4.51773** | **3.89E-05** |
| Right Lingual gyrus | -0.39735 | 0.770161 |  | **-2.83099** | **0.006319** |  | **-3.74707** | **0.000421** |
| Left Superior occipital gyrus | -1.0742 | 0.448292 |  | **-3.92591** | **0.000264** |  | **-3.71031** | **0.000592** |
| Right Superior occipital gyrus | -1.13135 | 0.416714 |  | **-3.97269** | **0.000232** |  | **-3.62866** | **0.000613** |
| Left Middle occipital gyrus | -0.78344 | 0.601361 |  | **-4.05509** | **0.000264** |  | **-5.0852** | **5.43E-06** |
| Right Middle occipital gyrus | -0.87013 | 0.5444 |  | **-3.7481** | **0.000613** |  | **-5.29036** | **2.65E-06** |
| Left Inferior occipital gyrus | -0.41061 | 0.770161 |  | **-3.85915** | **0.00031** |  | **-4.05633** | **0.000168** |
| Right Inferior occipital gyrus | 1.285826 | 0.349134 |  | **-2.46601** | **0.018077** |  | **-4.39647** | **5.54E-05** |
| Left Fusiform gyrus | -2.00124 | 0.173623 |  | **-6.01541** | **7.49E-08** |  | **-7.35375** | **1.27E-09** |
| Right Fusiform gyrus | -0.70086 | 0.631591 |  | **-4.90949** | **7.83E-06** |  | **-5.6592** | **7.16E-07** |
| Left Postcentral gyrus | -0.44751 | 0.765589 |  | **-3.25084** | **0.002593** |  | **-3.55211** | **0.000771** |
| Right Postcentral gyrus | -0.26648 | 0.846588 |  | **-3.1631** | **0.002676** |  | **-4.27656** | **8.20E-05** |
| Left Superior parietal gyrus | -2.273 | 0.150275 |  | **-4.64432** | **2.47E-05** |  | **-5.17214** | **4.04E-06** |
| Right Superior parietal gyrus | -1.28096 | 0.349134 |  | **-2.9378** | **0.004824** |  | **-4.36706** | **6.07E-05** |
| Left Inferior parietal gyrus | -0.29975 | 0.839285 |  | **-3.03193** | **0.003697** |  | **-5.01041** | **7.15E-06** |
| Right Inferior parietal gyrus | -1.51489 | 0.269077 |  | **-2.90681** | **0.005232** |  | **-4.33799** | **6.64E-05** |
| Left Supramarginal gyrus | -0.28414 | 0.842141 |  | **-3.47009** | **0.001077** |  | **-4.88952** | **1.04E-05** |
| Right Supramarginal gyrus | -0.22915 | 0.867224 |  | **-3.64494** | **0.000613** |  | **-4.10782** | **0.000145** |
| Left Angular gyrus | -1.06342 | 0.448292 |  | **-3.70558** | **0.000507** |  | **-3.84134** | **0.000317** |
| Right Angular gyrus | -3.19369 | 0.090638 |  | **-4.45225** | **4.61E-05** |  | **-5.62849** | **7.66E-07** |
| Left Precuneus | -2.09016 | 0.150275 |  | **-5.80868** | **8.53E-07** |  | **-6.69581** | **1.55E-08** |
| Right Precuneus | -1.61125 | 0.264491 |  | **-4.40474** | **9.44E-05** |  | **-5.0012** | **7.20E-06** |
| Left Paracentral lobule | 0.688237 | 0.632722 |  | **-2.57573** | **0.012761** |  | **-3.29888** | **0.001646** |
| Right Paracentral lobule | -0.86677 | 0.5444 |  | **-3.76333** | **0.000427** |  | **-4.17755** | **0.000114** |
| Left Caudate nucleus | -3.00305 | 0.090638 |  | **-4.41595** | **5.18E-05** |  | **-4.56407** | **3.41E-05** |
| Right Caudate nucleus | -2.3333 | 0.150275 |  | **-3.19572** | **0.002514** |  | -1.96638 | 0.055275 |
| Left Lenticular nucleus, Putamen | -2.40815 | 0.128893 |  | **-4.23203** | **9.44E-05** |  | **-5.82492** | **3.97E-07** |
| Right Lenticular nucleus, Putamen | -2.08457 | 0.150275 |  | **-2.51404** | **0.014952** |  | **-3.95276** | **0.000224** |
| Left Lenticular nucleus, Pallidum | 0.870904 | 0.5444 |  | -0.07915 | 0.937031 |  | -1.85044 | 0.072166 |
| Right Lenticular nucleus, Pallidum | 0.395373 | 0.770161 |  | -0.35138 | 0.734021 |  | **-2.12327** | **0.038955** |
| Left Thalamus | -2.14481 | 0.150275 |  | **-4.39548** | **5.45E-05** |  | **-5.58086** | **8.63E-07** |
| Right Thalamus | -1.70909 | 0.228902 |  | **-4.26984** | **8.61E-05** |  | **-5.41721** | **1.61E-06** |
| Left Heschls gyrus | -0.45654 | 0.765589 |  | **-4.46734** | **4.49E-05** |  | **-5.91807** | **2.96E-07** |
| Right Heschls gyrus | -0.13381 | 0.921384 |  | **-2.83191** | **0.006319** |  | **-3.77328** | **0.00039** |
| Left Superior temporal gyrus | -1.61741 | 0.264491 |  | **-4.98476** | **6.50E-06** |  | **-4.82791** | **1.30E-05** |
| Right Superior temporal gyrus | -2.40317 | 0.128893 |  | **-6.00471** | **7.49E-08** |  | **-6.4834** | **3.37E-08** |
| Left Temporal pole: superior temporal gyrus | -2.11204 | 0.150275 |  | **-4.92027** | **7.83E-06** |  | **-7.86014** | **2.20E-10** |
| Right Temporal pole: superior temporal gyrus | -1.42596 | 0.300831 |  | **-5.58318** | **8.96E-07** |  | **-5.26043** | **5.31E-06** |
| Left Middle tempooral gyrus | -2.13673 | 0.150275 |  | **-6.72172** | **3.18E-08** |  | **-6.45586** | **3.51E-08** |
| Right Middle tempooral gyrus | -2.14838 | 0.150275 |  | **-6.80265** | **4.33E-09** |  | **-7.24026** | **1.56E-09** |
| Left Temporal pole: middle temporal gyrus | -1.41117 | 0.300831 |  | **-3.14711** | **0.002713** |  | **-5.86916** | **3.46E-07** |
| Right Temporal pole: middle temporal gyrus | -2.61764 | 0.107382 |  | **-6.4007** | **1.57E-08** |  | **-6.28625** | **6.51E-08** |
| Left Inferior temporal gyrus | -2.22935 | 0.150275 |  | **-6.46247** | **1.39E-08** |  | **-7.3029** | **1.35E-09** |
| Right Inferior temporal gyrus | -2.15981 | 0.150275 |  | **-6.79002** | **4.33E-09** |  | **-6.63825** | **6.03E-08** |

ROI, regions of interest; aMCI, amnestic mild cognitive impairment; ADD, Alzheimer’s disease dementia;
